# Changes in serum markers failed to predict persistent infection after two-stage exchange arthroplasty

**DOI:** 10.1186/s13018-020-01923-z

**Published:** 2020-09-04

**Authors:** Qiao Jiang, Jun Fu, Wei Chai, Li-bo Hao, Yong-Gang Zhou, Chi Xu, Ji-Ying Chen

**Affiliations:** 1 Medical School of Chinese PLA, Beijing, China; 2 Department of Orthopedic Surgery, The First Medical Center, Chinese PLA General Hospital, Beijing, China; 3 Medical College, Nankai University, Tianjin, China; 4 General Hospital of People’s Liberation Army, No.28 Fuxing Road, Haidian District, Beijing, 100853 China

**Keywords:** Two-stage exchange arthroplasty, Value change, Percent change, Serum markers, Prosthetic joint infection

## Abstract

**Background:**

The proper timing of reimplantation is importation to treatment success in the two-stage exchange revision. The 2018 International Consensus Meeting suggested that a variation trend toward normalization in serum markers was useful for determining the proper timing of reimplantation. However, the opposite results were found by previous studies, and the normalization of serum markers was reported to fail to predict infection control. We investigated whether value changes and percent changes in four common serum markers (erythrocyte sedimentation rate (ESR), C-reactive protein (CRP), interleukin-6 (IL-6), and fibrinogen) can predict persistent infection.

**Methods:**

A retrospective review of 141 patients treated with the two-stage revision from 2014 to 2018 was conducted. The variation trend in serum indicators was evaluated by the percent changes (using values of serum markers pre-reimplantation divided by values pre-resection) and value changes (using values of serum markers pre-resection minus values pre-reimplantation). Treatment success was defined according to the Delphi-based consensus criteria with a minimum follow-up of 1 year, and the receiver operator characteristic (ROC) was used to examine the usefulness of changes in serum markers.

**Results:**

Twenty-two patients (15.60%) were persistently infected. No significant difference was found in either the value change or percent change in serum markers between reinfection and non-reinfection patients. When predicting persistent infection, the area under the curves (AUC) demonstrated that both percent changes and value changes in serum markers were poor indicators. The AUC of value changes was 0.533 for the CRP, 0.504 for the IL-6, 0.508 for the ESR, and 0.586 for fibrinogen when predicted persistent PJI. In addition, the AUC indicated that percent changes in the CRP (0.464), the IL-6 (0.534), the ESR (0.527), and fibrinogen (0.586) were all poor markers.

**Conclusions:**

We have shown that both value changes and percent changes in serum markers were not sufficiently rigorous to aid in persistent infection diagnosis. The proper timing of reimplantation must, therefore, take into account various clinical tests rather than the downward trend of serum markers only.

## Background

The management of prosthetic joint infection (PJI) is a challenging problem for clinicians, with a high prevalence after total joint arthroplasty (TJA) [1, 2]. In North America and East Asia, two-stage exchange arthroplasty is widely applied for chronic PJIs after TJA [3, 4]. After removing the infected prosthesis and implanting an antibiotic-loaded spacer in the first stage, the proper timing of reimplantation is crucial for successful treatment [5]. Currently, there is no “gold standard” to evaluate the eradication of PJI before reimplantation. The combination of serum indicators, synovial white blood cell (WBC) counts, culture results, intraoperative histology, and clinical symptoms is widely used to guide the timing of reimplantation.

The elevated erythrocyte sedimentation rate (ESR) and C-reactive protein (CRP) are minor criteria in MSIS criteria for predicting PJI [6–11]. However, the reliability and utility of both indicators have been questioned by several studies. Due to their unclear threshold cutoff levels, the normalization of both markers was reported to fail to predict PJI control [5–7, 11]. More than the ESR and CRP, several other serum biomarkers have been studied by researchers. Interleukin-6 (IL-6) was suggested to be useful in diagnosing PJI [12, 13], and Hoell et al. reported the high utility of IL-6 in predicting reimplantation failure [14]. Li et al. reported that fibrinogen had a good performance in the diagnosis of PJI [15]. However, more research is needed to determine the accuracy and reliability of these serum indicators for predicting the proper timing of reimplantation.

Instead of a numerical threshold, the suggestion that a variation trend toward normalization in serum markers was useful for determining the proper timing of reimplantation was with the approval of the majority in the 2018 International Consensus Meeting. However, the opposite result was found by Stambough et al. [16]. Stambough et al. [16] found that the area under the receiver operator curves (AUC) was 0.530 for the percent or delta change of the ESR and 0.482 for the change of CRP when predicting persistent PJI, indicating that both were poor markers. Considering the value of fibrinogen and IL-6 in diagnosing PJI, changes in these serum markers may be useful for predicting reinfection after reimplantation. In addition, more than percent changes, the downward trend in serum markers can be expressed as value changes and the usefulness of the value changes in serum markers should be investigated.

As contradictory results exist regarding whether changes in serum markers can guide the timing of reimplantation in two-stage exchange arthroplasty after total joint arthroplasty (TJA), we investigated (1) whether percent or delta changes in serum indicators, which included the erythrocyte sedimentation rate (ESR), C-reactive protein (CRP), interleukin-6 (IL-6), and fibrinogen are useful for predicting infection eradication; and (2) whether value changes in these four serum markers can guide the proper timing of reimplantation.

## Methods

### Patients

After receiving Institutional Review Board approval, we retrospectively reviewed all patients who underwent two-stage reimplantation between 2014 and 2018 (*n* = 161). All patients were confirmed to have chronic PJI, and acute hematogenous and perioperative infections were excluded [17]. The exclusion criteria were as follows: (1) incomplete recordings of serologic markers at the time of resection or reimplantation; and (2) less than the minimum 1-year follow-up or no reinfection occurrence within this period. In this study, the MSIS criteria [18] were considered the gold standard reference for diagnosing PJI before resection, and patients who did not meet the MSIS criteria were excluded. There was a total of 141 patients (81 hips and 60 knees) in the final analysis with complete records of serum biomarkers, and all patients met the MSIS criteria when diagnosed with PJI.

We reviewed all medical records of patients in detail, which included sex, age, gender, joint, American Society of Anesthesiologists (ASA) score, risk factors, surgical history of the same site, pathology results, and organism culture results, the details of which are summarized in Table 1.

**Table 1:** Patient demographics

	Non-reinfection (*n* = 119)	Reinfection (*n* = 22)	*p* value
Patient characteristics			
Follow-up (year)	3.45 ± 1.58	3.62 ± 1.90	0.240
Age (year)	58.44 ± 13.28	64.50 ± 11.07	0.162
BMI (kg/m^2^)	25.04 ± 3.60	24.92 ± 3.31	0.800
Interval of spacer (weeks)	23.39 ± 17.03	26.00 ± 27.82	0.380
Male	53 (44.54%)	12 (54.55%)	0.387
Hip	69 (57.98%)	12 (54.55%)	0.764
Sinus	42 (35.29%)	11 (50.00%)	0.191
Comorbidities			
Diabetes	16 (13.45%)	3 (13.64%)	0.981
Previous revision history	25 (21.01%)	6 (27.27%)	0.515
ASA ≥3	11 (9.24%)	3 (13.64%)	0.527
Inflammatory disease	13 (10.92%)	3 (13.64%)	0.713

*BMI* body mass index, *ASA* American Society of Anesthesiologists

### Treatment protocol

All patients underwent an institutional standard two-stage exchange arthroplasty, including the removal of the prosthesis, placement of an antibiotic-loaded articulating cement spacer, and thorough debridement at the time of the first stage procedure. Vancomycin (2–4 g per 40 g) and meropenem (1–2 g per 40 g) were mixed in polymethylmethacrylate (PMMA) spacers. Four to six samples for aerobic, anaerobic, and fungal culture and there to five samples for histology analysis were obtained intraoperatively from the periprosthetic membrane and other periprosthetic tissues in which infection was suspected. After the insertion of cement, all patients received 6–8 weeks of intravenous antibiotics depending on culture sensitivity reports. For patients with culture-negative results, broad-spectrum antibiotic therapy was used.

At least a 2-week antibiotic holiday was stipulated before reimplantation. Prior to implantation, joint aspiration was routinely performed in patients suspected of infection. The determination of proper timing to perform reimplantation was based on the combination of clinical symptoms, serum markers, and synovial tests. During the second-stage revision, the antibiotic-loaded cement was removed. Sterilized saline water (4–6 L) was used to irrigate the joint after thorough debridement. Three to five samples were sent for frozen sectioning, and aerobic and anaerobic cultures according to the surgeons’ suspicion.

The values of four main serum biomarkers including serum ESR, CRP, fibrinogen, and IL-6 were determined before resection and reimplantation. We used STA-R Evolution® analyzer (Stago Diagnostica, Asnieres, France) to gauge fibrinogen levels and expressed in gram per liter (g/L) [19]. The ESR was measured by the Westergren method, and CRP and IL-6 were gauged by nephelometric immune assay s[20]. The threshold values were 30 mm/h for ESR and 10 mg/L for CRP according to the MSIS criteria [18], and the upper limit was 12 pg/mL for IL-6 and 4.01 mg/mL for fibrinogen [15, 21] when diagnosing PJI. The variation trend toward normalization in serum indicators was evaluated by the percent changes (using values of serum markers at the time of reimplantation divided by values at the time of resection) and value changes (using values of serum markers at the time of resection minus values at the time of reimplantation).

### Definition of persistent PJI and treatment success

Persistent or recurrent PJI in this study was defined as (1) re-infection after reimplantation, based on MSIS criteria; (2) the presence of a sinus tract communicating with the joint at reimplantation; and (3) two positive intraoperative periprosthetic cultures with the same organism at reimplantation.

We determined treatment success using the Delphi-based criteria [22, 23], which matches the following: (1) a healed wound without fistula, drainage, pain, or infection recurrence caused by the same organism strain; (2) no subsequent surgical intervention for infection after reimplantation surgery; (3) no occurrence of PJI-related mortality.

### Statistical analysis

Categorical data were summarized as absolute values and percentage. The absolute values and changes (percent changes and value changes) in these four serum markers were presented as the median and interquartile range (IQR). The demographic and clinical characteristics between groups were compared with the use of the Student’s *t* test if they were normally distributed or the Mann-Whitney test if not normally distributed for continuous variables and the chi-square test or Fisher’s exact test for categorical variables. Receiver operating characteristic (ROC) curves were generated to determine the diagnostic value of each test for the assessment of persistent PJI. The area under the curve (AUC) was calculated. Discriminatory value of ROC curves was interpreted as excellent (AUC 0.9–1), good (0.8–0.89), fair (0.7–0.79), poor (0.6–0.69), or fail/no discriminatory capacity (0.5–0.59) [24]. Youden’s J-statistic was used to attempt to determine a threshold value for each serologic marker. A *p* value less than 0.05 was considered significant. SPSS version 20 (SPSS Inc., Chicago, Illinois) was used for statistical analysis.

## Results

### General information and patients’ follow-up

Demographic information and follow-up results are shown in Table 1. There were 54 (49.5%) males in the non-reinfection group and 12 (54.55%) in the reinfection group. The mean age was 58.44 ± 13.28 years in the success group and 64.50 ± 11.07 years in the re-infected group. The mean body mass index (BMI) was 25.04 ± 3.60 kg/m^2^ in the success group compared with 24.92 ± 3.31 kg/m^2^ in the reinfection group. Eighty-one hips and sixty knees were included in the analysis. There was no significant difference in the prevalence of sinus occurrence between the reinfection group (50.00%) and the non-reinfection group (35.29%, *p* = 0.191).

The mean follow-up year was 3.45 (range 1.32 to 8.70 years) years in the success group and 3.62 (range 0.65 to 7.32 years) years in the re-infected group. The interval of spacer insertion was 23.39 (range 4.57 to 110.86 weeks) weeks in the success group and 26.00 (range 5.29 to 143.71 weeks) weeks in the reinfection group. There were 22 patients re-infected after reimplantation in two-stage exchange arthroplasty, and the total success rate was 84.40%.

### Organism analysis

Details of the causative organisms in 22 infection patients are shown in Table 2. Coagulase-negative Staphylococcus (CNS) species were the most common causative bacteria for recurrent PJI (6, 27.27%). The rest of the causative organisms included 1 (4.55%) *Staphylococcus aureus*, 1 (4.55%) *Enterococcus faecalis*, 1 (4.55%) Gram-negative *Bacillus*, 1 methicillin-resistant Staphylococcus aureus (MRSA), 5 (22.73%) polymicrobial organisms, and 2 (9.09%) other organisms. Five (22.73%) patients had negative culture results but were diagnosed with PJI according to the MSIS criteria at the time of reinfection.

**Table 2:** Causative organisms of 22 persistent or recurrent PJI

Culture organism	Frequency	Percent
Staphylococcus aureus	1	4.55%
Coagulase negative Staphylococcus	6	27.27%
Gram-negative bacillus	1	4.55%
MRSA	1	4.55%
Enterococcus	1	4.55%
Polymicrobial organisms	5	22.73%
Other organisms	2	9.09%
Negative culture	5	22.73%

### Was there any difference in values of serum markers between the success and reinfection groups?

The values of the ESR, IL-6, CRP, and fibrinogen were compared between success patients and reinfection patients. The details of each serum marker are shown in Table 3. No significant difference was found in these four serum markers between the success group and the reinfection group at the time of resection and reimplantation. At the time of resection, the median CRP was 18.00 mg/L (IQR 7.95–35.50 mg/L) in the success group versus 25.50 mg/L (IQ, 16.89–38.18 mg/L) in the reinfection group (*p* = 0.087), and the median ESR was 35 mm/h (IQR 23.00–58.00 mm/h) for those patients who remained infection-free versus 48 mm/h (IQR 30.25–75.75 mm/h) for reinfection patients (*p* = 0.124). At the time of reimplantation, the median CRP was 3.48 mg/L (IQR 2.03–8.40 mg/L) in the success group versus 3.32 mg/L (IQR 1.60–15.30 mg/L) in the reinfection group (*p* = 0.643), and the median ESR was 12.00 mm/h (IQR 7.00–18.00 mm/h) for those patients who remained infection-free versus 12.00 mm/h (IQR 7.00–42.50 mm/h) for reinfection patients (*p* = 0.214).

**Table 3:** Values of serum markers at the time of resection and reimplantation in non-reinfection group and reinfection group

Serum markers	Non-reinfection (*n* = 119)			Reinfection (*n* = 22)			*p* value
	Median	25%	75%	Median	25%	75%	
Pre-resection							
CRP (mg/l)	18.00	7.95	35.50	25.50	16.89	38.18	0.087
IL-6 (pg/ml)	12.10	8.13	19.70	17.21	8.51	27.59	0.249
ESR (mm/h)	35.00	23.00	58.00	48.00	30.25	75.75	0.124
Fibrinogen (g/l)	4.82	4.09	5.44	5.40	4.56	6.19	0.607
Pre-reimplantation							
CRP (mg/l)	3.48	2.03	8.40	3.32	1.60	15.30	0.643
IL-6 (pg/ml)	3.42	2.00	5.43	3.34	2.00	14.54	0.379
ESR (mm/h)	12.00	7.00	18.00	12.00	7.00	42.50	0.214
Fibrinogen (g/l)	3.34	3.05	3.89	3.74	2.90	4.63	0.228

### Can the percent changes or value changes in serum markers guide the timing of reimplantation?

A comparison of percent changes and value changes in the non-reinfection group versus the reinfection group was shown in Table 4. No significant difference was found in either the value change or percent change in these four serum markers. When comparing pre-resection with pre-reimplantation values, the median value changes in the CRP was 11.95 mg/L (IQR 2.01–28.31 mg/L) in the non-reinfection group and 17.58 mg/L (IQR 0.45–36.61 mg/L) in the reinfection group (*p* = 0.627). The median value changes in the IL-6 was 7.91 pg/L (IQR 4.26–13.76 pg/L) for those patients who remained infection-free and 8.81 pg/L (IQR 3.55–15.56 pg/L) for those who experienced reinfection (*p* = 0.948). The median value changes in the fibrinogen were 1.32 g/L (IQR 0.69–1.95 g/L) in the infection-free group versus 1.85 g/L (IQR 0.37–2.95 g/L) in the reinfection group (*p* = 0.200). In addition, the median value changes in the ESR was 25.00 mm/h (IQR 9.00–40.00 mm/h) for those patients who remained infection-free and 26.00 mm/h (IQR 5.75–45.50 mm/h) for those who are re-infected (*p* = 0.901).

**Table 4:** Value changes and percent changes of serum markers between resection and reimplantation in reinfection and non-reinfection patients

Serum markers	Non-reinfection (*n* = 119)			Reinfection (*n* = 22)			*p* value
	Median	25%	75%	Median	25%	75%	
Value changes							
CRP (mg/l)	11.95	2.01	28.31	17.58	0.45	36.61	0.627
IL-6 (pg/ml)	7.91	4.26	13.76	8.81	3.55	15.56	0.948
ESR (mm/h)	25.00	9.00	40.00	26.00	5.75	45.50	0.901
Fibrinogen (g/l)	1.32	0.69	1.95	1.85	0.37	2.95	0.200
Percent changes (%)							
CRP	29.99	11.12	74.81	16.04	7.02	107.32	0.595
IL-6	29.58	16.37	55.39	36.45	16.00	55.56	0.611
ESR	30.77	16.85	61.90	29.91	20.49	78.31	0.687
Fibrinogen	71.66	61.69	83.50	63.03	54.03	92.08	0.242

With regard to percent changes from the resection to the reimplantation, the median percent change in the CRP was 29.99% (IQR 11.12–74.81%) for those patients who remained infection-free and 16.04% (IQR 7.02–107.32%) for those who experienced reinfection (*p* = 0.595). The median percent changes in the ESR was 30.77% (IQR 16.85–55.39%) in the non-reinfection group and 29.91% (IQR 20.49–78.31%) in the reinfection group (*p* = 0.611). The median value changes in the fibrinogen were 71.66% (IQR 61.69–83.50%) in the infection-free group versus 63.03 (IQR 54.03–92.08%) in the reinfection group (*p* = 0.242). In addition, the median value changes in the IL-6 was 29.58% (IQR 16.37–55.39%) for those patients who remained infection-free and 36.45% (IQR 16.00-55.56%) for those who are re-infected (*p* = 0.611).

**Fig. 1:**
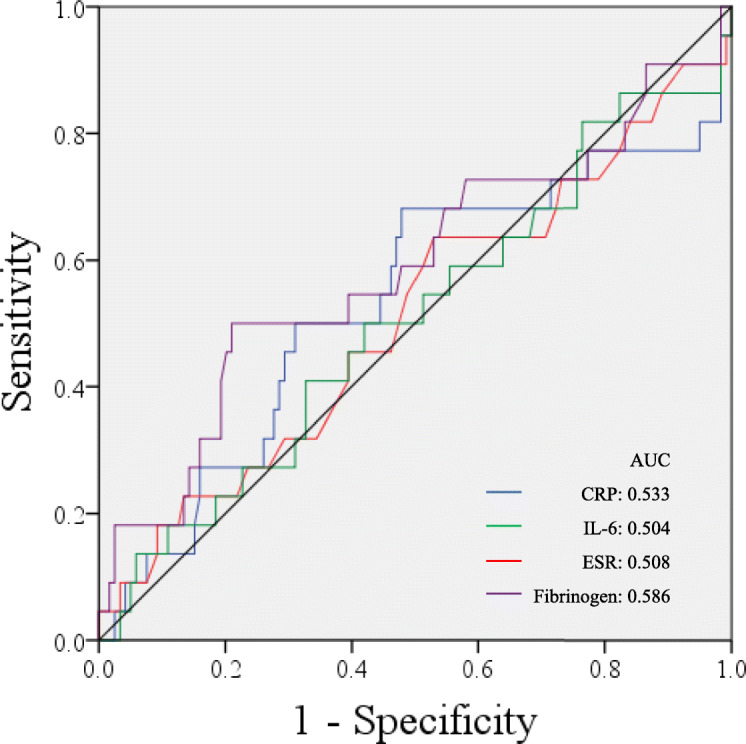
Receiver operator curves for value changes in serum markers in predicting failure after two-stage exchange arthroplasty: C-reactive protein (blue line), interleukin-6 (green line), erythrocyte sedimentation rate (red line), and fibrinogen (purple line). The black slope depicts 50% sensitivity and specificity

**Fig. 2:**
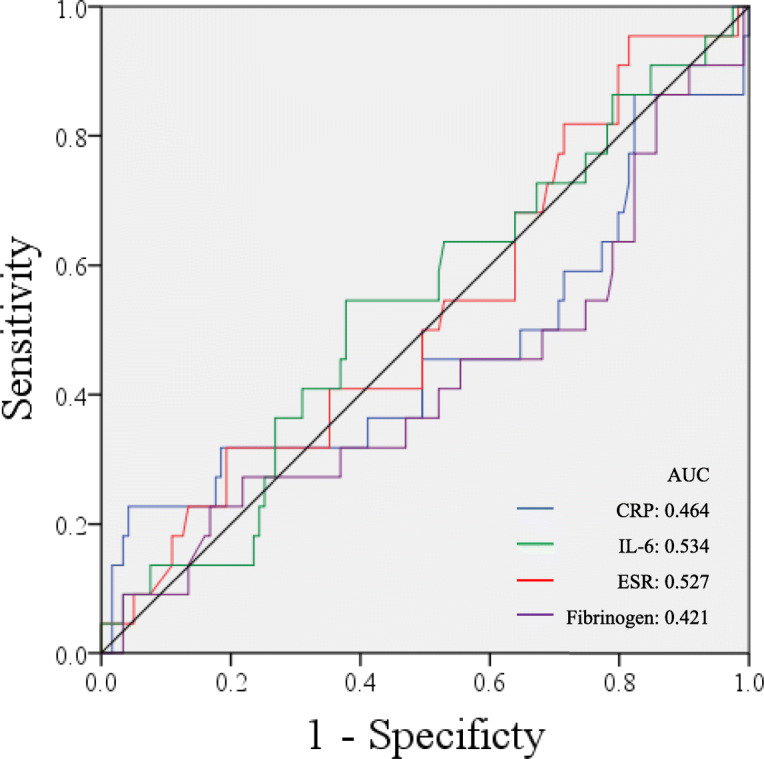
Receiver operator curves for percent change in serum markers in predicting failure after two-stage exchange arthroplasty: C-reactive protein (blue line), interleukin-6 (green line), erythrocyte sedimentation rate (red line), and fibrinogen (purple line). The black line depicts 50% sensitivity and specificity

Furthermore, we generated a receiver operator curve (ROC) for value changes (Fig. 1) and percent changes (Fig. 2) in these four serum markers. The area under the receiver operator curve (AUC) was 0.533 for CRP, 0.504 for IL-6, 0.508 for the ESR, and 0.586 for fibrinogen for predicting failure based on value changes, demonstrating that value changes in these four serum markers were poor indicators. With regard to percent changes for determining the timing of reimplantation, the AUC indicated that percent changes in the CRP (AUC = 0.464), the IL-6 (AUC = 0.534), the ESR (AUC = 0.527), and the fibrinogen (AUC = 0.586) were all poor markers when predicted persistent or recurrent infection. As a result of the low sensitivity and specificity caused by the wide distribution of changes in inflammatory levels, a threshold value could not be calculated by Youden’s J-statistic.

## Discussion

PJI is still a challenging problem after total joint arthroplasty, and two-stage exchange arthroplasty has been proven to be a useful treatment for prosthetic joint infection after total joint arthroplasty, with a success rate ranging from 65 to 100% [25]. The proper timing of reimplantation is crucial to prosthesis survival after reimplantation. Considering the unreliability of clinical symptoms, delayed pathology results, and scarcity of synovial fluid, serum biomarkers still play an important role in predicting persistent infection after reimplantation.

However, in our study, no significant difference was found in the median values of these four serum markers between the non-reinfection group and the reinfection group. This is not surprising given that several authors have failed to determine the threshold of the ESR and CRP. Kusuma et al. [6] reported 76 PJIs after total knee arthroplasty, and the AUC was 0.62 for the ESR and 0.39 for CRP. Qu et al. [26] reported the AUC of IL-6 was 0.59 when the threshold was set at 8.12 pg/mL. Xu et al. [19] also investigated 109 hips in patients who underwent two-stage exchange arthroplasty. They found that the fibrinogen had high specificity but low sensitivity when predicted persistent infection, and the threshold of fibrinogen was 3.61 g/L.

Instead of a numerical threshold value, the International Consensus Meeting recommended that the downtrend of serological tests and results of the synovial analysis should be used to determine the optimal timing of reimplantation. However, two traditional serum markers (the ESR and CRP) were studied by Stambough et al. [26], and the AUC for the percent change in Stambough et al. was 0.530 for the ESR and 0.482 for CRP. Ghanem et al. [7] studied a consecutive series of 109 patients who underwent two-stage resection arthroplasty for infected TKA. The total success rate was 79% in their cohort. The value change of the ESR and CRP was depicted by the ROC, and the AUC was 0.503 for the ESR and 0.545 for CRP. Both Stambough et al. and Ghanem et al. came to conclusions that opposed the ICM recommendations. We did, however, include the change of IL-6 and fibrinogen in our analysis. We found that both value changes and percent changes in these four laboratory markers were poor indicators for predicating re-infection after two-stage exchange reimplantation. A variation trend toward normalization in serum markers was not significantly associated with the eradication of infection, and this result may change the traditional beliefs of surgeons.

Infection eradication is not necessarily accompanied by the normalization of serum markers. Kusuma et al. [6] retrospectively reviewed the serologies of 76 infected patients who underwent two-stage revision. They found that the ESR remained persistently elevated in 37 knees (54%), and the CRP remained elevated in 14 knees (21%) where the infection had been controlled. Similarly, Shukla et al. studied eighty-seven hips with infected total hip arthroplasty and found that the mean ESR, CRP, and synovial fluid white blood cell (WBC) count are differential decreased. The ESR remained elevated in 50 patients (62.5%) and the CRP remained elevated in 22 patients (27.5%) in non-reinfection patients. However, the synovial WBC count was promising markers with the AUC of 0.91 when predicted persistent infection. The ESR and CRP were not useful in the diagnosis and frequently failed to normalize even in patients without persistent infection. In our study, similar results were found in changes in the IL-6 and fibrinogen.

To date, the accurate diagnosis of the eradication of infection is still difficult for clinicians. As our study proved that changes in four common serum markers had low utility for predicting persistent PJI, other useful ways, such as new biomarkers, biochemistry, and histology, should be proposed to detect infection. The combination of clinical symptoms, the value of serum markers, frozen sections, and synovial fluid WBC counts is still the most reliable method for surgeons to determine the timing of reimplantation.

There were several limitations to our study. First, this was a retrospective study and certain biases of retrospective studies cannot be avoided. Although we reviewed most cases in the study, some mistakes may have existed. Second, because there is no “gold standard” for diagnosing persistent PJI after reimplantation, we combined culture result and follow-up result to identify re-infection. Thus, we constructed a broad definition for the failure of the 2-stage exchange procedure and the subsequent PJI surgery after reimplantation might be indicative of a new infection rather than a persistent PJI. Third, several surgeons in our hospital conducted the surgery. Though institutional guidelines for therapy have been approved, differences still exist in the management of patients. Fourth, it was a single-institution study with a limited sample size and, as a result, has limited external validity. Only 22 patients developed treatment failure during the follow-up, which might be an insufficient sample size. However, we set strict inclusion criteria. Thus, changes in inflammatory markers are only affected by the joint infection, and the statistical result may be reliable.

## Conclusion

We have shown that both value changes and percent changes in serum markers were not sufficiently rigorous to aid in persistent infection diagnosis. The proper timing of reimplantation must, therefore, take into account various clinical tests rather than the downward trend of serum markers only.

## Data Availability

We do not wish to share our data because of some of the patient’s data regarding individual privacy, and according to the policy of our hospital, the data could not be shared with others without permission.

## Abbreviations

PJI: Prosthetic joint infection
WBC: White blood cell
TJA: Total joint arthroplasty
ESR: Erythrocyte sedimentation rate
CRP: C-reactive protein
IL-6: Interleukin-6
MSIS: Musculoskeletal Infection Society
IQR: Interquartile range
BMI: Body mass index
AUC: Area under the curve
ROC: Receiver operating characteristic
CNS: Coagulase-negative Staphylococcus
MRSA: Methicillin-resistant Staphylococcus aureus
